# Self-reported recall and daily diary-recorded measures of weight monitoring adherence: associations with heart failure-related hospitalization

**DOI:** 10.1186/1471-2261-14-12

**Published:** 2014-01-31

**Authors:** Christine D Jones, George M Holmes, Darren A DeWalt, Brian Erman, Jia-Rong Wu, Crystal W Cene, David W Baker, Dean Schillinger, Bernice Ruo, Kirsten Bibbins-Domingo, Aurelia Macabasco-O’Connell, Victoria Hawk, Kimberly Broucksou, Michael Pignone

**Affiliations:** 1 Division of General Medicine and Clinical Epidemiology, University of North Carolina at Chapel Hill, Chapel Hill, NC, USA; 2 The Cecil G. Sheps Center for Health Services Research, University of North Carolina at Chapel Hill, Chapel Hill, NC, USA; 3 Department of Health Policy and Management, University of North Carolina at Chapel Hill, Chapel Hill, NC, USA; 4 School of Nursing, University of North Carolina at Chapel Hill, Chapel Hill, NC, USA; 5 Division of General Internal Medicine, Feinberg School of Medicine, Northwestern University, Chicago, IL, USA; 6 Division of General Internal Medicine and Center for Vulnerable Populations, Department of Medicine at San Francisco General Hospital, University of California San Francisco, San Francisco, CA, USA; 7 Olive View-UCLA Medical Center, UCLA School of Nursing, University of California, Los Angeles, CA, USA; 8 NRSA Primary Care Fellowship, Division of General Medicine and Clinical Epidemiology, University of North Carolina at Chapel Hill, 5034 Old Clinic Building, CB# 7110, Chapel Hill, NC 27599, USA

**Keywords:** Heart failure, Patient compliance, Monitoring, Physiologic

## Abstract

**Background:**

Weight monitoring is an important element of HF self-care, yet the most clinically meaningful way to evaluate weight monitoring adherence is uncertain. We conducted this study to evaluate the association of (1) self-reported recall and (2) daily diary-recorded weight monitoring adherence with heart failure-related (HF-related) hospitalization.

**Methods:**

We conducted a prospective cohort study among 216 patients within a randomized trial of HF self-care training. All patients had an initial self-care training session followed by 15 calls (median) to reinforce educational material; patients were also given digital scales, instructed to weigh daily, record weights in a diary, and mail diaries back monthly. Weight monitoring adherence was assessed with a self-reported recall question administered at 12 months and dichotomized into at least daily versus less frequent weighing. Diary-recorded weight monitoring was evaluated over 12 months and dichotomized into ≥80% and <80% adherence. HF-related hospitalizations were ascertained through patient report and confirmed through record review.

**Results:**

Over 12 months in 216 patients, we identified 50 HF-related hospitalizations. Patients self-reporting daily or more frequent weight monitoring had an incidence rate ratio of 1.34 (95% CI 0.24-7.32) for HF-related hospitalizations compared to those reporting less frequent weight monitoring. Patients who completed ≥80% of weight diaries had an IRR of 0.37 (95% CI 0.18-0.75) for HF-related hospitalizations compared to patients who completed <80% of weight diaries.

**Conclusions:**

Self-reported recall of weight monitoring adherence was not associated with fewer HF hospitalizations. In contrast, diary-recorded adherence ≥80% of days was associated with fewer HF-related hospitalizations. Incorporating diary-based measures of weight monitoring adherence into HF self-care training programs may help to identify patients at risk for HF-related hospitalizations.

## Background

Heart Failure (HF) causes significant morbidity and mortality. Multiple trials have shown that self-care training can reduce HF-related hospitalizations [1]. Weight monitoring is an important element of HF self-care that enables patients to monitor their volume status [2]. Weight gain can often be the first sign of volume overload in patients with HF; if such weight gains are treated promptly, clinically significant HF exacerbations can be avoided. In clinical practice, self-reported measures are frequently used to assess whether patients perform components of HF self-care, including weight monitoring [3-5]. However, the most clinically meaningful way to evaluate adherence to weight monitoring is unclear. Using weight monitoring adherence measures to determine which patients are optimally performing HF self-care could help identify which patients may benefit from more intensive HF self-care training and support.

In a prior case–control analysis, we identified that optimal diary-recorded adherence to weight monitoring (≥80% adherence) measured over seven days was associated with a lower odds of HF-related ED visits or hospitalizations [6]. However, tracking adherence to weight monitoring by diary may be cumbersome for both patients and providers, leading us to speculate how a self-reported measure of weighing frequency that more closely approximates what is commonly used in practice might compare to a diary-recorded measure of weight monitoring adherence. We conducted this study to compare the association of self-reported and diary-recorded measures of weight monitoring adherence with HF-related hospitalizations. Since the optimal frequency to define weight monitoring adherence has not been established, we performed additional analyses to explore thresholds for weight monitoring adherence.

## Methods

### Study design

We performed a prospective cohort study that was nested in the intensive self-care intervention arm of a randomized clinical trial that compared different levels of HF self-care training [7]. Details of this trial have been described previously and will be summarized here. Patients in this study had either diastolic or systolic HF with New York Heart Association (NYHA) class II-IV symptoms within the prior 6 months, adequate cognitive function, were on a loop diuretic, and were fluent in either English or Spanish. Patients were recruited between 2006 and 2009 from Internal Medicine and Cardiology clinics at the University of North Carolina (UNC) at Chapel Hill, Northwestern University, Olive View – UCLA Medical Center, and UCSF – San Francisco General Hospital. Institutional Review Boards (IRB) from UNC, Northwestern University, Olive View-UCLA, and UCSF approved the protocol and all patients provided informed consent. The investigation conformed to the principles outlined in the Declaration of Helsinki.

### Intervention description

All patients in the intensive self-care intervention arm received an in-person 40-minute education session followed by a series of educator calls (median of 15) over 1 year. The education session and calls reinforced the importance of daily weight monitoring, taking proper diuretic doses, medication adherence, salt reduction, and exercise. Most patients (72% of intervention arm patients) were taught how to adjust their diuretic doses based on their daily weights. Among the four educators who delivered the intervention, two were registered dieticians with experience counseling patients in clinical settings; the other two had bachelor’s degrees and experience working as health educators. The educators convened for a one-day training prior to enrollment and participated in weekly calls with an investigator to develop the educational protocol and ensure similar education delivery across sites.

All patients were provided a digital bathroom scale and a diary in which they were to record their daily weights. Patients were instructed to mail weight diaries back to their educator on a monthly basis in self-addressed, stamped envelopes. The educator reviewed the diaries to assess how well a patient was following the protocol and incorporated that information in their ongoing interactions with that patient. At the conclusion of the study period (12 months after enrollment), trained study personnel collected the diaries from each site and entered them into a central database for analysis.

The initial number of patients in the intensive self-care training arm was 303. For this analysis, we made exclusions for submitting no diary data during the trial (n = 67) or for a missing response to the self-reported weighing adherence measure from the 12 month survey (n = 20 additional exclusions), leaving a sample of 216 patients.

### Measures and data collection

A trained research assistant (RA) at each site administered baseline questionnaires and collected information about race/ethnicity, income, and insurance status. Socioeconomic status (SES) was measured with the MacArthur Scale of Subjective Social Status; this measure asks individuals to indicate on which ladder rung they feel that they stand relative to other people in the United States with regard to money, education, and jobs (range 0–9) [8]. HF severity was assessed using a single item with a series of structured statements to categorize patients by New York Heart Association (NYHA) class. HF-related quality of life was assessed using the Improving Chronic Illness Care Evaluation (ICICE) HF Symptom Scale (HFSS); [9] HFSS scores were transformed to a 100-point scale with 100 representing the least symptoms/best health. Literacy was measured with a Short Test of Functional health Literacy in Adults (s-TOFHLA) and categorized into adequate literacy (≥ 23 out of 36) or inadequate/marginal literacy (< 23 out of 36) [10]. The RA abstracted medical records to collect additional demographic and clinical data.

### Outcome assessment

The primary end-point for this study was HF-related hospitalization. The University of North Carolina Survey Research Unit conducted telephone surveys at 1, 6, and 12 months during which patients were asked about any hospitalizations or other events that occurred in the previous time period. Upon identification of a hospitalization, admission and discharge summaries were requested from the hospital; these records were reviewed by one of three adjudication committee members masked to intervention status to determine if the hospitalization was HF-related. Adjudication committee members used a study protocol and their clinical judgment to assess whether HF was present at admission and whether HF was an important contributing factor to the hospitalization. If the first assessor concluded that a hospitalization was definitely HF-related or definitely not HF-related, no further assessment was done. For all other hospitalizations, another assessor conducted a second assessment. If the first two assessors disagreed, a third assessor reviewed the hospitalization and the case was discussed by the full committee to resolve differences.

### Weight monitoring adherence: self-reported and diary-recorded

Weight monitoring adherence was measured using two methods: self-reported frequency of weighing and diary-recorded weights. Self-reported weight monitoring adherence was collected at baseline and during telephone interviews using the following question: “How often do you weigh yourself?” Patients who reported weighing at least once a day (“every day” or “twice a day or more”) were considered optimally adherent; those who reported weighing less frequently (“several times a week”, “once every week or two”, or “less frequently than every 1 to 2 weeks”) were considered sub-optimally adherent. This threshold was chosen because weighing “every day” indicates a stricter adherence level that is closer to our ≥80% diary threshold than “several days a week”, which could represent a wide range of adherence. Our main predictor variable in the self-reported weight monitoring adherence analysis was collected following completion of the full intervention at the 12-month interview. Diary-recorded weight monitoring adherence was determined from weight diaries and dichotomized into ≥80% and <80% days of adherence to weight monitoring over a patient’s days in the trial to reflect optimal and sub-optimal adherence, respectively. The ≥80% threshold was chosen because this threshold is commonly used to define optimal medication adherence, [11-13] and because we used this threshold to define weight monitoring adherence in a prior publication [6]. We also performed sensitivity analyses to evaluate how different thresholds of weight monitoring adherence were associated with HF-related hospitalizations.

### Statistical analysis

We compared demographic and clinical data by adherence groups for both self-reported and diary-recorded data for descriptive purposes with a chi-square test for dichotomous variables and Student’s t-test for continuous variables. A Fisher’s exact test was used to compare dichotomous variables with 5 or fewer individuals in 25% of categories. A Kolmogorov-Smirnov test was used to assess for normal distribution of continuous variables; for continuous variables found to have a non-normal distribution, a Wilcoxon-Mann–Whitney test was used to compare groups.

We compared the incidence rates of HF hospitalizations between (1) self-reported optimal versus sub-optimal adherence groups and (2) diary-recorded optimal versus sub-optimal adherence groups using negative binomial regression. In one negative binomial regression model we adjusted only for site; in the fully adjusted negative binomial regression model, we included demographic and clinical factors that were either statistically significantly different between groups in bivariate analyses (i.e., site, age, race/ethnicity, SES, HF QOL, previous MI/angina, self-reported weighing at baseline, and systolic dysfunction) or have been previously associated with HF-related hospitalizations (i.e., gender) [14]. Standard errors were adjusted for clustering by site. Of note, we did not include total educator calls in the final model because educator calls were likely to have been part of the causal pathway in the association between weight monitoring adherence and HF-related hospitalizations.

We performed sensitivity analyses in which we used different adherence thresholds for diary-recorded weight monitoring adherence (i.e., ≥90%, ≥70%, and ≥60% thresholds). We considered a two-sided p value of <0.05 statistically significant. All data analyses were performed using Stata 12.0 (College Station, TX).

## Results

### Patient characteristics

Among 216 patients the mean age was 62.2 years, about half were male, and 40% were African American (Table 1). Nearly half of patients reported an income < $15,000/year, 34% had inadequate literacy, and approximately half had NYHA Class II HF. Of interest, only 28% of patients reported weighing daily or more frequently at baseline. The 87 patients excluded from this analysis because they either submitted no diary data during the trial (n = 67) or a were missing a 12 month self-reported weighing adherence measure (n = 20) were more likely to be younger (58.4 years), male (63%), have NYHA Class III or IV HF (42%), and were less likely to have a prior MI or angina (25%).

**Table 1:** Baseline characteristics of individuals: optimal and Sub-optimal weight monitoring adherence from self-reported and diary-recorded measures

**Characteristics**	**Overall sample** **(N = 216)**	**Self-reported weighing daily or more (N = 184)**	**Self-reported weighing less than daily (N = 32)**	**P value**	**Diary-recorded ≥80% weighing adherence (N = 107)**	**Diary-recorded <80% weighing adherence (N = 109)**	**P value**
Age (mean, SD)	62.2 (13.9)	62.4 (13.9)	61.1 (13.5)	0.638	64.3 (12.6)	60.1 (14.8)	0.024
Men (N, %)	103 (48)	83 (45)	20 (63)	0.069	45 (42)	58 (53)	0.101
Race/Ethnicity							
White, Non-Hispanic	80 (37)	71 (39)	9 (28)	0.024	47 (44)	33 (30)	0.071
Hispanic	35 (16)	24 (13)	11 (34)		12 (11)	23 (21)	
African American	87 (40)	76 (41)	11 (34)		43 (40)	44 (40)	
Other	14 (6)	13 (7)	1 (3)		5 (5)	9 (8)	
NYHA Class (N, %)							
I	45 (21)	39 (21)	6 (19)	0.536	23 (21)	22 (20)	0.168
II	111 (51)	96 (52)	15 (47)		61 (57)	50 (46)	
III	35 (16)	27 (15)	8 (25)		15 (14)	20 (18)	
IV	25 (12)	22 (12)	3 (9)		8 (7)	17 (16)	
Study site (N, %)							
UNC	87 (40)	80 (43)	7 (22)	<0.001^a^	56 (52)	31 (28)	<0.001
Northwestern University	66 (31)	62 (34)	4 (13)		33 (31)	33 (30)	
UCSF	39 (18)	22 (12)	17 (53)		6 (6)	33 (30)	
Olive view, UCLA	24 (11)	20 (11)	4 (13)		12 (11)	12 (11)	
Socioeconomic status, median (IQR)	4 (3, 6)	5 (3,7)	4 (1, 5)	0.026^b^	4 (2, 6)	4 (3, 6)	0.455^b^
Inadequate literacy (N, %)	74 (34)	59 (32)	15 (47)	0.103	30 (28)	44 (40)	0.06
Heart failure related QOL: Heart failure symptom scale, median (IQR)	61 (43, 79)	61(46, 79)	59 (43, 75)	0.272^b^	64 (46, 82)	57 (43, 71)	0.014^b^
Income level							
<$15,000	101 (47)	79 (43)	22 (69)	0.052	43 (40)	58 (53)	0.098
$15,000-$24,999	40 (19)	34 (18)	6 (19)		20 (19)	20 (18)	
>$25,000	71 (33)	67 (36)	4 (12)		41 (38)	30 (28)	
Previous MI/Angina (N, %)	83 (38)	76 (41)	7 (22)	0.037	49 (46)	34 (31)	0.027
Systolic dysfunction (N, %)	123 (57)	112 (61)	11 (34)	0.005	65 (61)	58 (53)	0.263
ACE-Inhibitor or ARB (N, %)	178 (82)	154 (84)	24 (75)	0.233	86 (80)	92 (84)	0.437
Beta-Blocker (N, %)	176 (81)	153 (83)	23 (72)	0.130	90 (84)	86 (79)	0.324
Thiazide	22 (10)	18 (10)	4 (13)	0.750	10 (9)	12 (11)	0.823
Spironolactone	50 (23)	46 (25)	4 (13)	0.172	22 (21)	28 (25)	0.425
Telephone calls with educator, median (IQR)	16 (14, 18)	16 (15, 18)	15 (13, 16)	<0.001^b^	17 (16, 18)	15 (13, 17)	<0.001^b^
Self-reported weighing daily or more at baseline, (N, %)	61 (28)	58 (32)	3 (9)	0.005^a^	35 (33)	26 (24)	0.148
Taught weight-based diuretic self-adjustment	178 (82)	152 (83)	26 (81)	0.852	87 (81)	91 (83)	0.674

^a^Groups compared using Fisher’s exact test.

^b^Groups compared using both t-test and Wilcoxon-Mann–Whitney test.

NYHA: New York Heart Association; MI: Myocardial Infarction; ACE: Angiotensin-Converting Enzyme; ARB: Angiotensin Receptor Blocker; IQR: Interquartile Range; QOL: Quality of Life.

### Weight monitoring adherence: self-reported and diary-recorded

At 12 months, 85% of patients completing a survey (184 of 216) self-reported weighing themselves at least daily (Table 1). Those self-reporting weighing daily or more were more likely to be White, Non-Hispanic (39% versus 28% reporting less than daily, p 0.024), have a higher SES (score of 5 versus 4, p 0.026) , report previous MI or angina (41% versus 22%, p 0.037), systolic dysfunction (61% versus 34%, p 0.005), and on average completed one more telephone call with the clinical educator than patients who reported weighing less than daily (16 calls versus 15 calls, p <0.001, Table 1).

Over 12 months, 50% of patients (107 of 216) recorded and returned ≥80% of daily weights. Those who recorded ≥80% of weights were more likely to be older (64.3 versus 60.1 years for <80% weighing adherence, p 0.024), report a higher HF quality of life (score of 64 versus 57 of 100, p 0.014), report previous MI or angina (46% versus 31%, p 0.027), and on average completed two more telephone calls with the clinical educator than patients who recorded <80% of daily weights (17 versus 15 calls, p <0.001, Table 1). Those who were taught weight-based diuretic self-adjustment were distributed evenly among all groups.

### Weight monitoring adherence and HF hospitalization

Among 216 patients, we identified 50 HF-related hospitalizations over 12 months; 20 patients had 1 hospitalization, 8 patients had 2 hospitalizations, and 4 patients had 3 or more HF-related hospitalizations. Patients with optimal self-reported weight monitoring adherence at 12 months had 0.26 events per person-year (47 events in 184 patients) and those self-reporting sub-optimal adherence had 0.09 events per person-year (3 events in 32 patients). Patients with ≥80% diary-recorded weight monitoring adherence had 0.14 events per person-year (15 events in 107 patients) and those with <80% diary-recorded weight monitoring adherence had 0.32 events per person-year (35 events in 109 patients).

Table 2 shows incidence rate ratios (IRR) for HF hospitalizations both for self-reported and diary-recorded weight monitoring adherence. Ten patients were missing one or more covariate and were excluded from the adjusted models (adjusted model N = 206). In those with optimal self-reported weight monitoring adherence, the IRR for HF hospitalization 1.99 (95% CI 0.69-5.72) adjusted only for study site and 1.34 (95% CI 0.24-7.32) in a fully adjusted model; neither result was statistically significant. In those with diary-recorded weight monitoring adherence of ≥80%, we found a statistically significant lower incidence of HF hospitalization, both in a model adjusted only for study site (IRR 0.32, 95% CI 0.15-0.70) and in a fully adjusted model (IRR 0.37, 95% CI 0.18-0.75).

**Table 2:** Association of weight monitoring adherence with HF hospitalizations, self-reported and diary-recorded

**Models**	**N**	**Incidence rate ratios**	**95% confidence intervals**
**Self-reported daily or more weight monitoring adherence (at 12 months)**			
Adjusted for site only	216	1.99	(0.69-5.72)
Overall adjusted^a^	206	1.34	(0.24-7.32)
**Diary-recorded weight monitoring adherence (≥ 80% over 12 months)**			
Adjusted for site only	216	0.32	(0.15-0.70)
Overall adjusted^a^	206	0.37	(0.18-0.75)

^a^Model adjusted for age, gender, race, site, subjective SES, HF quality of life, previous MI/angina, systolic dysfunction, and self-reported weight monitoring adherence (daily or more) at baseline.

### Sensitivity analyses

**Figure 1:**
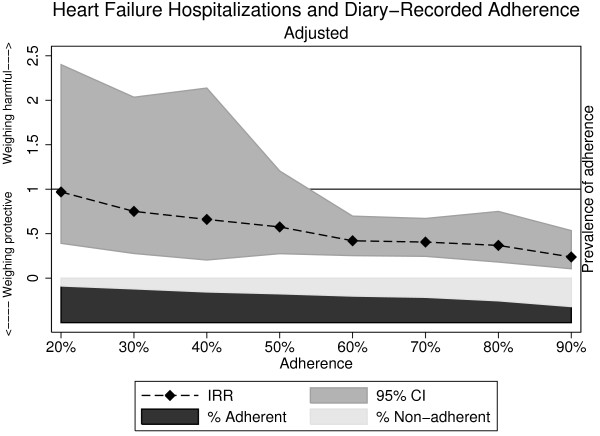
**Association of heart failure-related hospitalization with diary-recorded adherence over 12 months, adjusted incidence rate ratio**^**a**^**. **^a^Model adjusted for age, gender, race, site, subjective SES, HF quality of life, previous MI/angina, systolic dysfunction, and self-reported weight monitoring adherence (daily or more) at baseline.

When we changed the diary-recorded optimal adherence threshold to ≥60%, ≥70%, and ≥90%, the magnitude of effect strengthened with each step up in adherence (Table 3). Thresholds for adherence that ranged down to ≥60% were associated with a lower risk for HF-related hospitalization that was statistically significant. Figure 1 demonstrates the association between HF-related hospitalization and diary-recorded adherence as a continuous measure over 12 months; of note, confidence intervals widened considerably with diary-recorded adherence less than 60%, which was related to smaller sample sizes. To address potential selection bias that may have been introduced by excluding patients who never submitted a diary, we performed sensitivity analyses including this group and found similar IRRs for HF-related hospitalizations when we adjusted only for study site, both for the self-reported recall measure (IRR 1.92, 95% CI 0.80-4.62) and for the diary-recorded measure of adherence (IRR 0.24, 95% CI 0.14-0.41).

**Table 3:** Association of weight monitoring adherence with HF hospitalizations, sensitivity analyses

**Models**	**Incidence rate ratios**	**95% confidence intervals**
**Diary-recorded adherence: varying adherence thresholds**		
≥ 90%^a^	0.24	(0.11-0.54)
≥ 80% (main analysis)^a^	0.37	(0.18-0.75)
≥ 70%^a^	0.41	(0.25-0.67)
≥ 60%^a^	0.42	(0.25-0.70)

^a^Model adjusted for age, gender, race, site, subjective SES, HF quality of life, previous MI/Angina, systolic dysfunction, and self-reported weight monitoring adherence (daily or more) at baseline.

## Discussion

In this study, we found that patients self-reporting at least daily weight monitoring adherence did not have reduced rates of HF hospitalization compared to those self-reporting less frequent weighing adherence at 12 months. In contrast, we found that patients with ≥80% diary-recorded weight monitoring adherence had a statistically significantly lower rate of HF hospitalization than those with <80% adherence over 12 months of follow-up. Our findings suggest that diary-recorded measures of adherence may accurately identify optimal self-care that is associated with a reduced risk of HF-related hospitalizations.

HF self-care behaviors are frequently assessed through global self-reported measures, including the Self-Care of HF Index (SCHFI), [3] the revised Heart Failure Self-Care Behavioral Scale [15,16], the European HF Self-Care Behavioral Scale (EHFScB Scale), [4] and the Revised HF Compliance scale, [5,17] all of which contain an item to assess weighing behavior. Better HF care/behavior scores on respective indices/scales have been associated with improved clinical outcomes [17,18]. In studies that have specifically evaluated the association between weight monitoring adherence and clinical outcomes, the association between adherence and clinical outcomes has been variable based on the adherence measure used [17,19,20].

Daily weight monitoring is a mainstay of HF self-care, yet the most clinically meaningful measure to evaluate adherence to this behavior is not clear. Comparisons have been made between measures that are self-reported and measures obtained from repeated sampling of current behaviors or symptoms over a time period, termed “ecological momentary assessment” (EMA), in the work of Shiffman and colleagues [21]. EMA measures have the potential to “minimize recall bias, maximize ecological validity, and allow study of microprocesses that influence behavior in real-world contexts” (pg 1) [21]. EMA can be collected many ways, including diaries, telephone calls, and electronic records. The main objective of EMA is to ascertain multiple samples of symptoms or behaviors experienced at the time the sample is collected. Self-reported and EMA measures have been found to be discrepant in studies from various populations, including patients with alcoholism, [22] tobacco use, [23] urinary incontinence, [24] and headaches [25].

Discrepancies between self-reported and EMA measures of medication adherence [e.g., medication event monitoring systems (MEMS) caps (medication bottle caps that record times/dates that bottles are opened)], have also been noted in patients with HF [19,20]. Such discrepancies between adherence measures can be attributed to a number of characteristics, such as the frequency with which measures are collected, the duration of time over which measures estimate behavior, the types of behaviors being evaluated, and other factors [21]. Overall, self-reported measures are at risk for systematic recall bias when compared to EMA measures [21].

In our study, measures used to assess daily weight monitoring adherence fall into the general categories of self-reported recall and EMA from a daily diary. We found that a self-reported recall of optimal adherence at 12 months was associated with a somewhat higher risk of HF-related hospitalizations that was not statistically significant; the small size of the group self-reporting optimal adherence likely contributed to less precision in this outcome. Yet, we did find an association between diary-recorded weight monitoring adherence and HF-related hospitalizations at multiple thresholds of adherence, with an effect size that strengthened with increasing thresholds. Adherence thresholds ranging down to ≥60% of days were associated with decreased risk for HF-related hospitalization, suggesting benefit even at thresholds lower than our pre-specified cut-point of ≥80%.

One may speculate whether completion of weight diaries in this study is merely a reflection of program engagement and/or more advanced HF self-care skills/knowledge. To evaluate whether program engagement may have explained some of this association, we added the number of educator calls into our adjusted model and found that this did not lead to attenuation of the IRRs from our main analyses (results not shown). We theorize this is because the number of educator calls may have varied based on a range of factors including: a patient’s engagement in the program, lack of mastery of educational material that required more calls, or even patient-initiated calls due to concerns about their regimen or symptoms. Of note, the weight diaries in this study were used to teach a majority of patients (72% of intervention arm patients) how to self-adjust their diuretics based on weight. Although the observed association could have been partly related to this aspect of the intervention, the proportion of patients who were taught diuretic self-adjustment did not differ significantly by adherence in either self-reported or diary-recorded groups (Table 1).

### Limitations

With regard to study limitations, our self-reported adherence question did not specify the duration of time for which patients were to report weighing behavior, which likely increased recall bias in responses. In addition, given that 85% of patients self-reported weighing at least daily at 12 months, patient responses to this question may not have accurately reflected behavior, potentially due to social desirability or other factors. It is important to note that the surveys were delivered by masked outcome assessors. The smaller sample size and very low rate of HF-related hospitalization in the group that self-reported sub-optimal adherence contributed to less precision in our results and lack of statistically significant findings.

In addition, our measure of diary-recorded weight monitoring averaged adherence over the entire follow-up period. As such, it is possible that adherence directly preceding HF hospitalizations differed from overall adherence; however, in a prior case–control study nested in this same study population, we found that diary-recorded weight monitoring adherence (≥80%) in the week preceding HF emergency department visits or hospitalizations was similarly protective [6].

Our comparison between self-reported and diary-recorded adherence measures was not a direct comparison between randomized groups of individuals, but instead a comparison of how different measures of adherence were associated with clinical outcomes in the same group of individuals. We noted baseline differences between groups who were optimally adherent and sub-optimally adherent to both self-reported and diary-recorded measures; thus selection bias or unmeasured confounding may have affected our results. However, we attempted to mitigate such differences by adjusting for these variables in our analysis; in addition, we found comparable results to our main analysis when we performed a sensitivity analysis in which we included patients who never submitted a weight diary. The generalizability of our findings may be limited by our younger patients with less advanced HF than in other studies of HF self-care [1,26]. Strengths of this study include a diverse, well-characterized sample drawn from a multi-site trial and rigorous outcome assessment.

## Conclusions

In conclusion, we found that ≥80% adherence to a daily diary-recorded measure was associated with fewer HF hospitalizations, but that a self-reported recall measure of weight monitoring adherence was not associated with fewer HF hospitalizations. Diary-recorded weight monitoring adherence may identify more rigorous adherence to HF self-management skills as well as program engagement and may provide more clinically meaningful information than a self-reported recall measure.

## Competing interests

The authors declare that they have no competing interests.

## Authors’ contributions

CDJ conceived of the study, participated in design of study and drafted the manuscript; GMH participated in the design of the study and performed the statistical analysis; DAD conceived of the study, participated in design of study, acquisition of data, and provided key revisions to manuscript; BE participated in the design of the study and participated in performing statistical analyses; JRW participated in the design of the study and provided key revisions to the manuscript; CWC participated in the design of the study and provided key revisions to the manuscript; DWB participated in the design of the study, acquisition of data, and provided key revisions to the manuscript; DS participated in the design of the study, acquisition of data, and provided key revisions to the manuscript; BR participated in acquisition of data, and provided key revisions to the manuscript; KBR participated in acquisition of data and provided key revisions to the manuscript; AMO participated in the acquisition of data and provided key revisions to the manuscript; VH participated in the design of the study and acquisition of data; KB participated in the design of the study and acquisition of data; MP conceived of the study, participated in design of study, acquisition of data, and provided key revisions to manuscript. All authors read and approved the final manuscript.

## Authors’ information

Work performed at: The University of North Carolina at Chapel Hill, Northwestern University, The University of California at San Francisco, and Olive View-UCLA Medical Center.

## Pre-publication history

The pre-publication history for this paper can be accessed here:

http://www.biomedcentral.com/1471-2261/14/12/prepub
